# MoO_3_@MoS_2_ Core-Shell Structured Hybrid Anode Materials for Lithium-Ion Batteries

**DOI:** 10.3390/nano12122008

**Published:** 2022-06-10

**Authors:** Muhammad Faizan, Sajjad Hussain, Mobinul Islam, Ji-Young Kim, Daseul Han, Jee-Hwan Bae, Dhanasekaran Vikraman, Basit Ali, Saleem Abbas, Hyun-Seok Kim, Aditya Narayan Singh, Jongwan Jung, Kyung-Wan Nam

**Affiliations:** 1 Department of Energy & Materials Engineering, Dongguk University, Seoul 04620, Korea; faizijaff@gmail.com (M.F.); endend42@naver.com (D.H.); basitalikhan077@gmail.com (B.A.); aditya@dongguk.edu (A.N.S.); 2 Hybrid Materials Center (HMC), Sejong University, Seoul 05006, Korea; shussainawan@gmail.com (S.H.); jwjung@sejong.ac.kr (J.J.); 3 Department of Nanotechnology and Advanced Materials Engineering, Sejong University, Seoul 05006, Korea; 4 Advanced Analysis & Data Center, Korea Institute of Science and Technology (KIST), Seoul 02792, Korea; jykim1128@kist.re.kr (J.-Y.K.); jhbae@kist.re.kr (J.-H.B.); 5 Division of Electronics and Electrical Engineering, Dongguk University-Seoul, Seoul 04620, Korea; v.j.dhanasekaran@gmail.com (D.V.); hyunseokk@dongguk.edu (H.-S.K.); 6 Centre for Energy Storage Research, Korea Institute of Science and Technology (KIST), Seoul 02792, Korea; saleem.abbas203@gmail.com

**Keywords:** core-shell structure, hybrid anode, MoS_2_, MoO_3_, hydrothermal synthesis

## Abstract

We explore a phase engineering strategy to improve the electrochemical performance of transition metal sulfides (TMSs) in anode materials for lithium-ion batteries (LIBs). A one-pot hydrothermal approach has been employed to synthesize MoS_2_ nanostructures. MoS_2_ and MoO_3_ phases can be readily controlled by straightforward calcination in the (200–300) °C temperature range. An optimized temperature of 250 °C yields a phase-engineered MoO_3_@MoS_2_ hybrid, while 200 and 300 °C produce single MoS_2_ and MoO_3_ phases. When tested in LIBs anode, the optimized MoO_3_@MoS_2_ hybrid outperforms the pristine MoS_2_ and MoO_3_ counterparts. With above 99% Coulombic efficiency (CE), the hybrid anode retains its capacity of 564 mAh g^−1^ after 100 cycles, and maintains a capacity of 278 mAh g^−1^ at 700 mA g^−1^ current density. These favorable characteristics are attributed to the formation of MoO_3_ passivation surface layer on MoS_2_ and reactive interfaces between the two phases, which facilitate the Li-ion insertion/extraction, successively improving MoO_3_@MoS_2_ anode performance.

## 1. Introduction

For the ever-growing need for renewable resources, the energy storage area has attracted much attention to alleviate environmental catastrophe and energy difficulties caused by traditional fossil fuels [1]. In this regard, rechargeable Li-ion batteries (LIBs) have become by far the most promising energy storage systems for portable electronic devices and electric vehicles, due to their comparatively greater energy and power density, and long-cycle life [2,3,4]. Since the electrochemical properties of the active materials govern the LIB’s performance, the development of new materials for LIBs is a crucial factor. Although LIBs are receiving growing attention, a graphite anode with a theoretical capacity of 372 mAh g^−1^ cannot meet the challenging requirements for higher energy density [5].

To substitute for graphite, various anode materials have been explored to improve the electrochemical performance, including transition metal dichalcogenides (TMDs) [6,7,8,9,10], transition metal oxides (TMOs) [11,12,13], titanium-based materials [14,15], carbon nanomaterials [16,17], and their composite materials [18,19]. Alloying-type anodes (Si, Sn, Ge) are the other alternative candidates that can replace graphite due to their very high theoretical capacity and relatively higher working potential, beneficial for reducing the risk of dendrite formation. However, the major drawback of these alloy-type anode materials is their massive volume expansion during cycling: for instance, Si expands up to 320%, leading to the failure of the active material. Extensive studies are ongoing to fix their volume expansion and stabilize them for LIBs [20,21,22]. Anode materials of the insertion type, such as carbonaceous materials and titanium-based oxides, have limited specific capacity. Compared to these insertion-type materials, conversion-type materials have a higher theoretical capacity of (600–1000 mAh g^−1^) and may be ideal to meet the energy demand for large-scale energy storages [23]. Over the past several decades, TMOs, one of the conversion-type materials’ families, have been studied extensively in LIBs [23,24]. Recent research has shifted focus from transition metal oxides (TMOs) to transition metal sulfides (TMSs), due to their better electrical conductivity, mechanical and thermal stability, and higher electrochemical activity [25,26]. The majority of TMSs have 2D layered structures with a chemical composition of MS_n_ (where *n* = 1 or 2, and M = Mo, W, Ti,V, or Sn). With an analogous layered structure to that of graphite anodes, 2D TMSs enable high rate capability via facile Li insertion and extraction, while the strong covalent bonding within the layers provides structural rigidity and robustness, leading to stable cyclability [27]. Even the interlayer spacings of these layer-structured TMSs are wider than graphite (~3.3 Å), for example, ~6.2 Å for MoS_2_, ~5.9 Å for SnS_2_, and ~5.7 Å for VS_2_ [28]. Using 2D TMSs with larger interlayer spacing can significantly reduce the diffusion barriers for Li-ions, and accommodate their expansion upon insertion. Most importantly, TMSs have a higher voltage platform compared to graphite anodes, which prevents the formation of lithium dendrites, and ensures their high safety [26]. Thanks to those advantageous properties, several TMS-based materials have shown larger Li storage capacities than graphite anodes, making them promising candidates for high-performance LIB anodes [25,26]. Molybdenum sulfide (MoS_2_), one of the most intriguing TMSs, has been studied in a number of applications, including nanoelectronics, electrocatalysis, and energy storage systems [29,30]. The MoS_2_ anode in LIBs undergoes an intercalation-assisted conversion reaction, where a Mo nanocrystal is embedded in the Li_2_S matrix as the final lithiated product and displays a high theoretical capacity of 670 mAh g^−^^1^ [28,31].

Although TMSs materials have many advantages, there are still challenges that need to be urgently solved. A particular problem is the sulfur dissolution in electrolytes caused by soluble polysulfide intermediates that occur during Li_2_S formation/decomposition, which results in the loss of sulfur components, thus abruptly deteriorating the capacity [26]. Various composite strategies with transition metal oxides have been explored to address the above problems, such as the TiO_2_@MoS_2_ [32], SnO_2_/SnS_2_ [33], MoO_2_/MoS_2_ [34], MoO_3_/MoS_2_ [35], MoO_3_/SnS_2_ [36], Fe_2_O_3_/MoS_2_ [37], and MoS_2_/MnO [38], which showed improved electrochemical performance in LIBs. However, making composites with TMO materials would not be optimal for mitigating the severe shuttling of lithium polysulfide (LiPSs), since the sulfide electrode interface remains exposed to the electrolyte. Surface protection, such as introducing a metal oxide protective layer, might help reduce polysulfide shuttling by preventing the dissolution of intermediates and accumulation of the sulfur phase on the lithium counter electrode [39]. W.-H. Ryu et al. reported a WO_3_ protective layer on WS_2_ nanofiber anode material, which dramatically improves the cycling performance in a sodium-ion battery [40]. Therefore, the synergistic effect of structurally stable TMOs’ surfaces with conductive TMS cores can be the key characteristic of potentially excellent electrochemical performance.

Herein, motivated by such synergetic effects of the TMO-covered TMS hybrid structure, we have proposed an in situ formation of MoO_3_ on the MoS_2_ TMS (denoted as MoO_3_@MoS_2_) by simple post-annealing of hydrothermally synthesized MoS_2_. Structural characterizations have verified the formation of the MoO_3_@MoS_2_ hybrid structure. The MoO_3_@MoS_2_ hybrid anode has demonstrated much-improved cycling stability compared with the single-phase MoS_2_ and MoO_3_ counterparts, without sacrificing the high power and capacity.

## 2. Experimental Section

### 2.1. Synthesis of MoS_2_, MoO_3_@MoS_2,_ and MoO_3_

The modified one-pot method was employed to synthesize the MoS_2_ [41]. First, thiourea (SC(NH_2_)_2_, solution A), and ammonium molybdate ((NH_4_)_2_MoO_4_, solution B) were dissolved in separate beakers for 30 min, followed by pouring solution B into solution A. This mixture was stirred moderately for another 30 min. Next, the transparent solution was placed into a 100 mL capacity Teflon-lined, stainless-steel autoclave, which was transferred to a convection oven programmed for the reaction at 200 °C for 12 h. After natural cooling to room temperature (RT), the black precipitates were collected, centrifuged with DI water and ethanol three times, then vacuum-dried at 100 °C for 1 h. Then, the as-synthesized MoS_2_ was further calcined at (200, 250, and 300) °C at a 5 °C/min rate in a box furnace. As per the structural results, the (200, 250, and 300) °C annealed samples were denoted as MoS_2_, MoO_3_@MoS_2,_ and MoO_3_, respectively.

### 2.2. Material Characterizations

High-resolution powder X-ray diffraction (HRPD) data were collected at the 9B beamline of the Pohang Acceleration Laboratory (PAL, Pohang, South Korea), using a monochromated X-ray with a wavelength (λ) of 1.5183 Å. The Raman spectra were collected using a Renishaw inVia system with an excitation wavelength of 532 nm. X-ray photoelectron spectroscopy (XPS) measurements (Ulvac PHI X-tool spectrometer) were performed with Al–Kα X-ray radiation, to investigate the chemical and valance states of the materials. The Mo K-edge X-ray absorption spectroscopy (XAS) spectra were measured at the Pohang Accelerator Laboratory (PAL) 8C beamline, in transmission mode, and processed with Athena software. The morphology and microstructure of the powders were analyzed by field-emission scanning electron microscopy (FE-SEM, Regulus 8230, HITACHI) and transmission electron microscopy (TEM, Talos F200X, ThermoFisher). Thermogravimetric analysis (TGA) of the as-prepared sample was carried out on a NETZSCH (STA 449F3, Selb, Germany) thermal analyzer with a heating rate of 10 °C/min in open-air conditions. Electrical conductivity was evaluated using a Loresta-GP low-resistivity meter (MCP-T610, Mitsubishi Chemical corp.). The sample porosity characteristics were determined by nitrogen adsorption and desorption measurements at 77 k (Micromeritics, Norcross, GA, USA).

### 2.3. Electrochemical Measurements

The 2032-coin cells with a two-electrode configuration were used to perform the electrochemical experiments. The MoS_2_, MoO_3_@MoS_2_, and MoO_3_ powder samples were thoroughly mixed with conductive carbon black and poly(vinylidene) fluoride (PVDF) binder dissolved in N-methyl pyrrolidone (NMP) solution in an 8:1:1 weight ratio to make a viscous slurry. The working electrode was prepared using a doctor blade casting on a Cu foil current collector. The electrode loading level was controlled to be ~2.27 mg cm^−2^ of active material. We used a TMAX roll press machine for calendaring. The density of electrodes was calculated to be 1.8997 g/cm^3^. The coin cells were assembled inside a glove box filled with Ar gas, with a Li metal counter electrode, a separator (Celgard 3501), and 1.0 M LiPF_6_ in a 1:1 (v:v) ratio of ethylene carbonate/dimethyl carbonate with 3% FEC additive as the electrolyte. After stabilizing the coin cell for 24 h, electrochemical tests were performed using a Neware (CT-4008–5 V 10 mA-164) battery cycler at RT. A Bio-Logic electrochemical system (VMP-3) was used to measure the cyclic voltammetry (CV). The cells were cycled at different current densities (35 to 700) mA g^−1^ between the potential window of (0.01–3.00) V. A cyclic voltammetry (CV) test was done under a 0.1 mV s^−1^ scan rate within the potential window of (0.01–3.00) V. A PGSTAT 302 electrochemical workstation (Metrohm Autolab) was employed to measure the electrochemical impedance spectroscopy (EIS) in the frequency range (100 kHz to 0.01 Hz) at an open-circuit voltage (OCV).

## 3. Results and Discussion

**Scheme 1:**
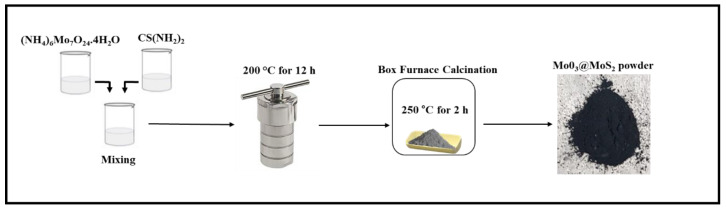
Schematic of MoO_3_@MoS_2_ synthesis.

**Figure 1:**
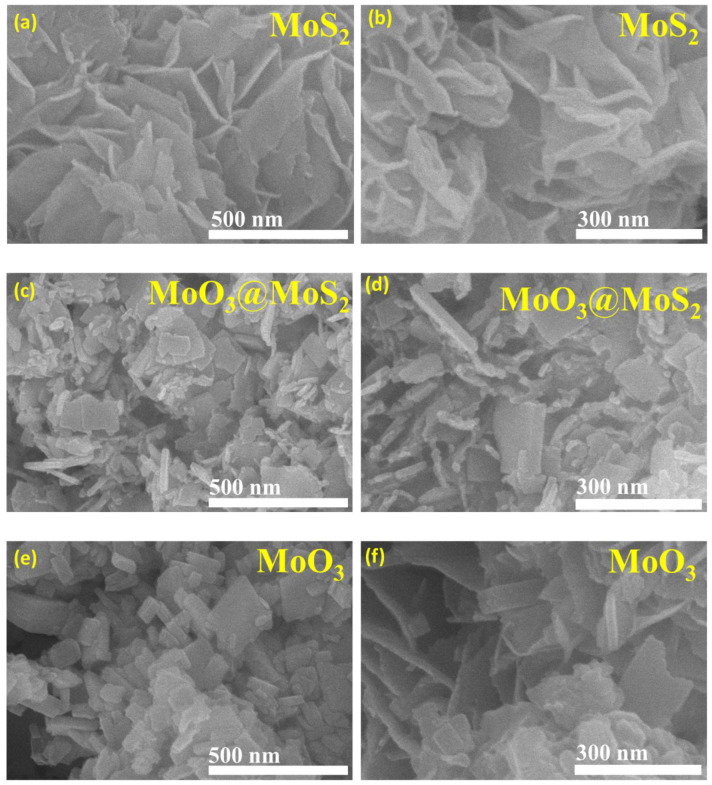
FE-SEM images for (**a**,**b**) MoS_2_, (**c**,**d**) MoO_3_@MoS_2_, and (**e**,**f**) MoO_3_.

Scheme 1 shows a schematic of the hydrothermal reaction followed by post-annealing in the air to synthesize MoO_3_@MoS_2_ hybrid material. The SEM images in Figure 1a,b demonstrate that the as-prepared MoS_2_ exhibits a 2D-sheet-like morphology, reflecting its 2D hexagonal structure, similar to the previous reports [27,41]. These nanosheets are twisted together due to their curved morphology, which causes many voids between them. When annealed at 250 °C in the air, the surface of the 2D-sheet-like structures appears somewhat disrupted, associated with the potential formation of a MoO_3_ layer (Figure 1c,d). The presence of Mo, S, and O in the EDS (Energy dispersive spectroscopy) pattern (Figure S1 of the Supplementary Information (SI)) indicates that the MoO_3_@MoS_2_ hybrid has been successfully formed. Evidently, the MoO_3_@MoS_2_ hybrids exhibit a morphology that shows a mixture of sheet-like and particle-like characteristics, such that the surfaces appear excessively rough, in apparent contrast to the smooth surface of the pristine MoS_2_ nanosheets (Figure 1a). On the other hand, when the sample is further annealed at 300 °C, different sizes of cuboid-shaped grains are observed for pure MoO_3_ formation (Figure 1e,f).

**Figure 2:**
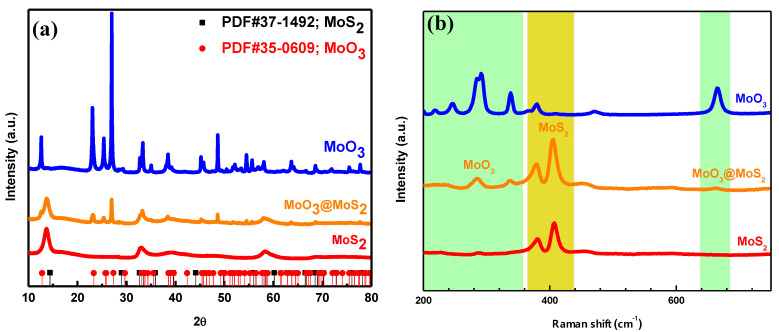
(**a**) HRPD and (**b**) Raman spectra of MoS_2_, MoO_3_@MoS_2_, and MoO_3_.

Synchrotron-based high-resolution powder diffraction (HRPD) was used to investigate the crystal structure of the MoS_2_, MoO_3_@MoS_2_, and MoO_3_ samples, as presented in Figure 2a and Figure S2. The characteristic peaks of the MoS_2_ sample annealed at 200 °C match very well with those of the hexagonal-structured 2D MoS_2_ with the space group *P*6_3_/*mmc* (JCPDS # 37–1492), while the intense XRD peaks for MoO_3_ annealed at 300 °C confirm the formation of a highly crystalline α-MoO_3_ phase with an orthorhombic structure and space group *Pbnm* (JCPDS # 35–0609). Notably, the MoO_3_@MoS_2_ sample annealed at 250 °C exhibits the diffraction peaks corresponding to both hexagonal MoS_2_ and orthorhombic MoO_3_ phases, verifying the hybrid structure formation. Notably, the MoO_3_ peak intensity in the MoO_3_@MoS_2_ hybrid was relatively lower than that of the MoO_3_ sample heated at 300 °C, suggesting the relatively lower content of the MoO_3_ than the MoS_2_. Figure S3a–c depicts the HRPD profile matching data of MoS_2_, MoO_3_@MoS_2_, and MoO_3_. The as-prepared hybrid material perfectly matches and contains the well-positioned diffraction peaks of MoS_2_ and MoO_3_. The lattice volume (v) of the MoS_2_ component in MoO_3_@MoS_2_ hybrid decreased by 0.71% when it was annealed at 250 °C, as shown in Table S1. The lattice volume contraction is due to the formation of the MoO_3_ layer, which compresses the core lattice [42]. In the hybrid, however, the MoS_2_ phase is not sufficiently crystallized to allow Rietveld refinement for calculating the weight fraction of MoS_2_ and MoO_3_.

Furthermore, the Raman spectra in Figure 2b also prove the successful formation of the MoO_3_@MoS_2_ hybrid, agreeing with the XRD results. The MoS_2_ spectrum presents the E^1^_2g_ (380.5 cm^−1^) and A_1g_ (406 cm^−1^) peaks corresponding to the in-plane and out-of-plane vibration modes of Mo-S, respectively [43]. The characteristic peak for the MoO_3_ phase positioned at 664 cm^−^^1^ belongs to B_2g_/B_3g_ mode associated with the triply coordinated oxygen stretching in the 3MoO^6−^ [44]. The Raman spectrum of the MoO_3_@MoS_2_ demonstrates the signature of both MoS_2_ and MoO_3_ peaks, confirming the formation of the MoO_3_@MoS_2_ hybrid.

**Figure 3:**
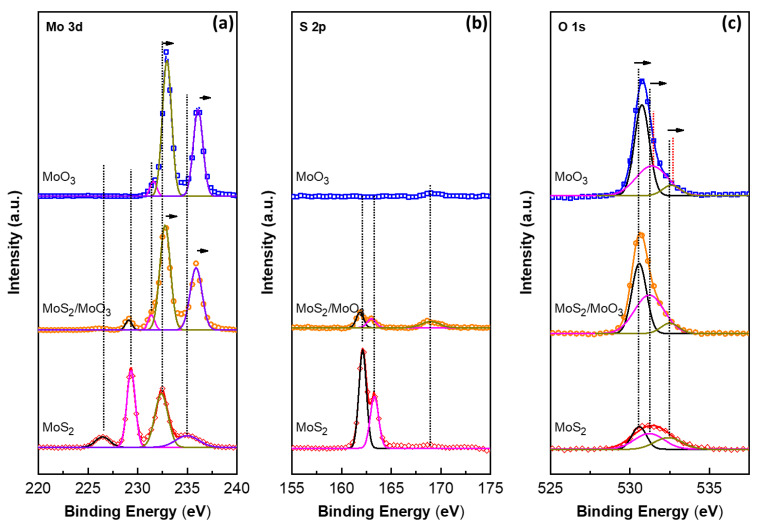
(**a**) Mo 3d, (**b**) S 2p, and (**c**) O 1s XPS spectra of MoS_2_, MoO_3_@MoS_2_, and MoO_3_.

XPS analysis was further performed to verify the chemical and electronic state at the sample’s surface. Figure S4 of the SI shows the survey spectrum of the MoO_3_@MoS_2_ sample, supporting the concurrent presence of Mo, S, and O elements in the prepared sample. Figure 3a compares the high-resolution Mo 3d core level XPS spectrum for the MoS_2_, MoO_3_@MoS_2_, and MoO_3_ samples. Two distinct peaks displayed at 229.3 and 232.5 eV for MoS_2_ correspond to Mo^4+^ 3d_3/2_ and 3d_5/2_ states, respectively. The high-resolution XPS spectrum of the MoO_3_ profile exhibits the 236.1 and 233.5 eV peaks corresponding to the Mo^6+^ 3d_3/2_ and 3d_5/2_ states, respectively [45]. The Mo 3d region of the MoO_3_@MoS_2_-XPS profile reveals the presence of mixed Mo^6+^ and Mo^4+^ oxidation states. Interestingly, the intensity of two distinct peaks at 236.1 and 233.5 eV associated with the MoO_3_ is predominant in the XPS spectra of the MoO_3_@MoS_2_ sample. Meanwhile, the two other peaks correspond to Mo^4+^ oxidation states associated with the MoS_2_ shift to low binding energy, suggesting that electron transfer occurs between MoO_3_ and MoS_2_ [37]. This finding implies that the hybrid sample shows a strong coupling between the MoS_2_ and MoO_3_ interface. Figure 3b shows the S 2p XPS region of MoS_2_, MoO_3_@MoS_2,_ and MoO_3_. For MoS_2_, the deconvoluted S 2p profile exhibits the doublet at 162.1 and 163.2 eV, while MoO_3_ shows no peak, confirming that only O and Mo are present in the MoO_3_ sample. The MoO_3_@MoS_2_ hybrid displays a noticeable shift of the two S 2p peaks to a lower binding energy, revealing that the MoO_3_ surface layer influences the valence states of the S species. The spectrum also includes a weak and broad peak at 168.9 eV, likely corresponding to the S-O bond formed during the post-annealing in air [46,47]. In Figure 3c, the deconvoluted O 1s region clearly establishes the Mo-O bonding in the MoO_3_ and MoO_3_@MoS_2_ hybrid samples [48,49,50]. Environmental sources and complete oxidation at 300 °C are attributed to the observed weak and strong O 1s peaks of MoS_2_ and MoO_3_, respectively. When compared to the MoO_3_@MoS_2_ hybrid sample, the shallow O 1s peak supports the evolution of the MoO_3_ oxide layer at the MoS_2_ surface.

**Figure 4:**
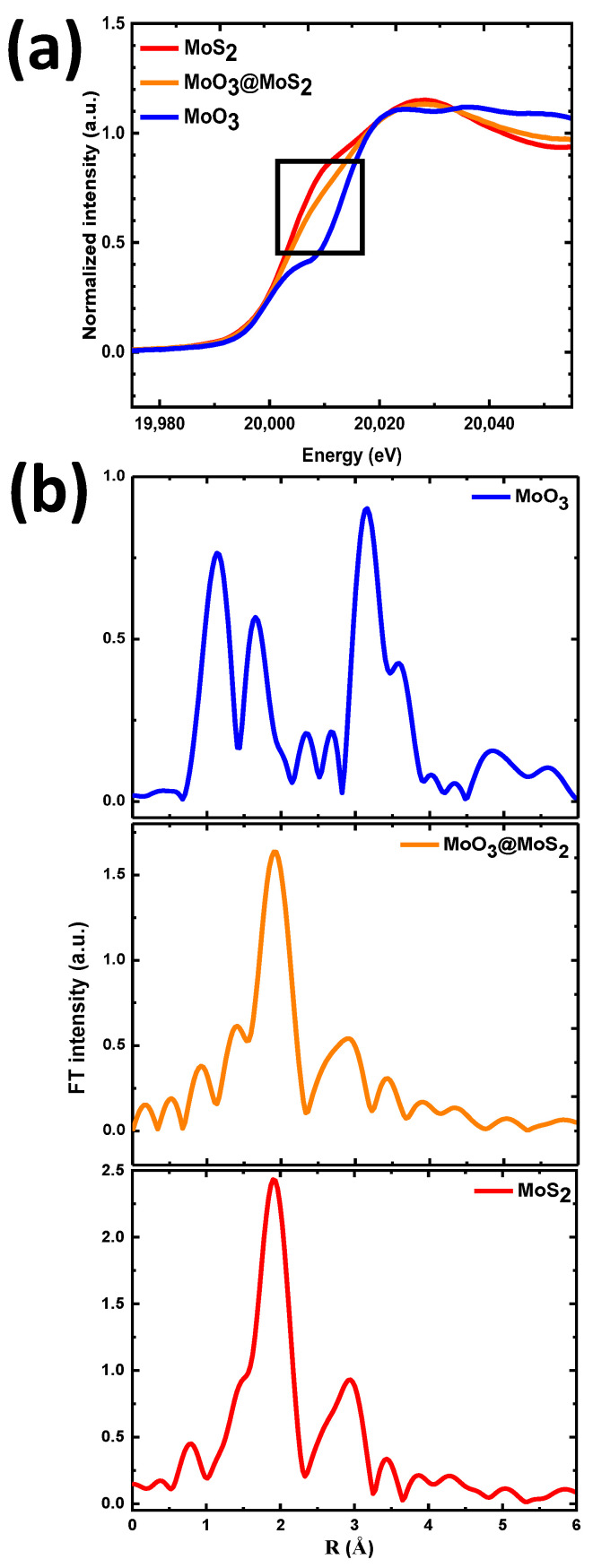
Mo K-edge (**a**) XANES, and (**b**) FT-EXAFS spectra, of MoS_2_, MoO_3_@MoS_2_, and MoO_3_.

**Scheme 2:**
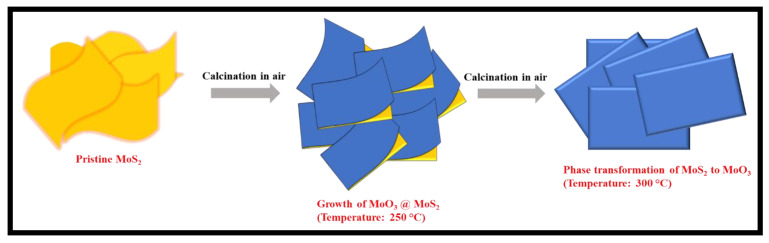
Schematic of the phase transformation of MoS_2_ between (~200 and ~300) °C.

Overall, the Raman results are in complete accordance with the XRD results, but XPS results show somehow opposite behavior regarding the crystal structure of the MoO_3_@MoS_2_ hybrid sample. While XRD is a bulk probe (detected in the micrometer range), XPS is a surface probe (of a few nanometers). Based on the XPS data recorded in the Mo 3d and S 2p regions, the peak density associated with the sulfur phase dropped while the peak density for the oxide phase strengthened (Figure 3a,b), suggesting the MoS_2_ surface almost converted to MoO_3_. XRD results show that the MoS_2_ phase predominates over the MoO_3_ phase in bulk, while the XPS peaks show that the oxide phase prevails on the surface of the MoO_3_@MoS_2_ hybrid. The ratio of the integral area between oxygen and sulfur obtained from XPS survey data is 9:1 (O2p/S2p~90.42/9.58). This ratio asserts our statement that the surface of MoS_2_ almost converted to MoO_3_. In contrast, we observed 1.5 wt% loss after the oxidation process above 250 °C in the TGA curve of as-synthesized MoS_2_ (Figure S5). Based on the calculation (see the SI), the bulk MoO_3_:MoS_2_ ratio is approximately 1.5:8.5 in the MoO_3_@MoS_2_ hybrid. This finding confirms the results of XRD and Raman. Furthermore, another bulk probe technique, X-ray absorption spectroscopy (XAS), also exhibits a similar tendency to XRD. The Mo K-edge position of the MoO_3_@MoS_2_ hybrid is much closer to that of MoS_2_ spectra, as shown in Figure 4a. There are two notable peaks in the Fourier-transformed extended X-ray absorption fine structure (FT-EXAFS) spectra (without phase correction) of MoS_2_ at approximately 1.91 and 2.92 Å, corresponding to Mo-S and Mo-Mo interaction in the first and second coordination shells, respectively [51]. As expected, the FT-EXAFS spectrum of MoO_3_@MoS_2_ matches very well with MoS_2_, rather than MoO_3_. This finding further confirms that the MoS_2_ phase dominates the core of the MoO_3_@MoS_2_ hybrid, validating our XRD and Raman results. In light of these results, we conclude that the precise control of the post-annealing procedure (i.e., 250 °C in air) produces a MoO_3_ surface layer on the MoS_2_ backbone. Scheme 2 graphically presents the structural changes during the post-annealing. The optimization of the post-calcination temperature (~250 °C) is critical in tuning the surface oxidation degree of the MoO_3_@MoS_2_ hybrid, as the sulfide phase completely transforms into MoO_3_ at 300 °C.

**Figure 5:**
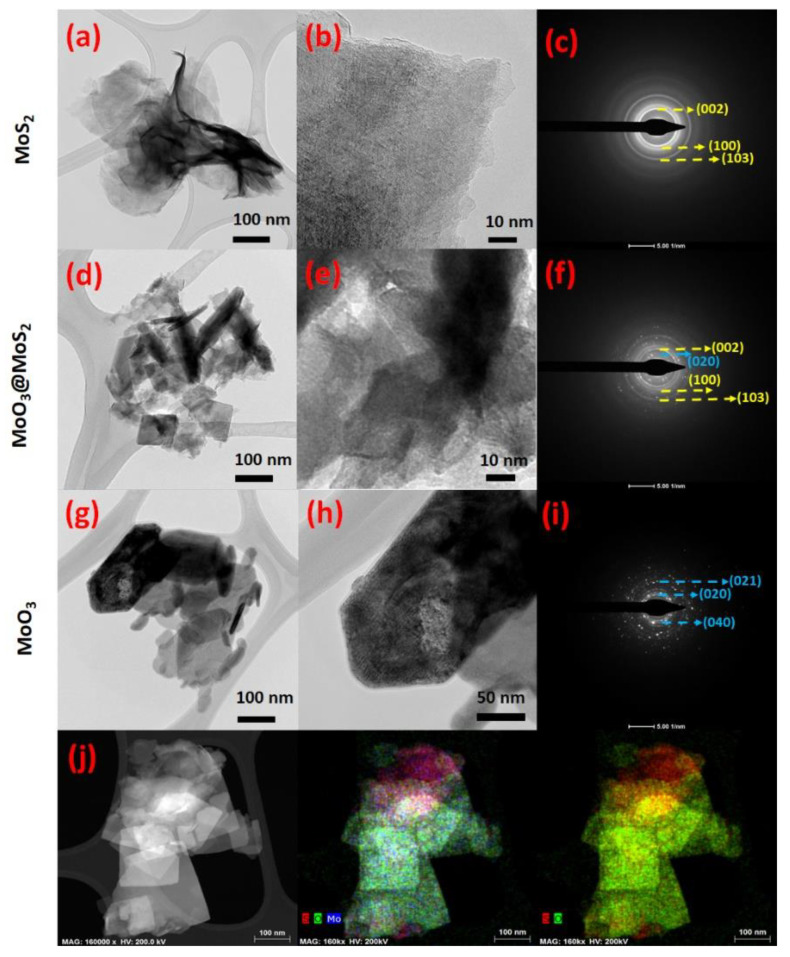
TEM images and corresponding SAED pattern of (**a**–**c**) MoS_2_, (**d**–**f**) MoO_3_@MoS_2_, (**g**–**i**) MoO_3_. (**j**) TEM-EDS mapping image of MoO_3_@MoS_2_.

The morphology and phase information of MoS_2_, MoO_3_@MoS_2_, and MoO_3_ nanostructures are further elucidated by TEM/EDS analysis, as depicted in Figure 5. The typical sheet-like morphology of MoS_2_ is further confirmed by TEM images (Figure 5a,b). The selected area electron diffraction (SAED) patterns in Figure 5c displays three diffraction rings for the (002), (100), and (103) lattice planes, indicating the hexagonal MoS_2_ polycrystalline structure (JCPDS # 37–1492). After introducing MoO_3_, the sheet-like structures of MoS_2_ are partially broken, as seen in Figure 5d,e. An enlarged TEM image shows d_002_ edges of the MoS_2_ phase (Figure S6 of the SI). The MoO_3_@MoS_2_ diffraction ring intensity appears weaker and blurrier than MoS_2_ on the SAED (Figure 5f), but likely relates to the MoS_2_ phase. This indicates that the sulfide phases are oxidized on the surface and turn into MoO_3_ phases, which disrupts the MoS_2_ crystallinity. These observations are consistent with the XRD and XPS results (Figure 2a and Figure 3). In comparison, the cuboid-shaped MoO_3_ (Figure 5g,h) shows entirely different SAED patterns (Figure 5i). The TEM-EDS elemental mapping (Figure 5j) indicates the uniform distribution of the Mo, S, and O species in the MoO_3_@MoS_2_ particles. It is evident that sulfur (red) exists within the particle’s core bodies, while oxide (green) is present at the surface, entirely covering the core. Figure S7a–c shows the BET sorption isotherm of MoS_2_, MoO_3_@MoS_2_, and MoO_3_ samples. The type-III isotherm profiles and absence of a hysteresis loop suggest the low porosity of all the materials. The BET surface area of MoS_2_, MoO_3_@MoS_2_, and MoO_3_ is only 8.71, 8.42, and 9.85 m^2^ g^−1^, respectively (Table S2). The pore distribution curve (Figure S7d) indicates the presence of only interparticle porosity.

**Figure 6:**
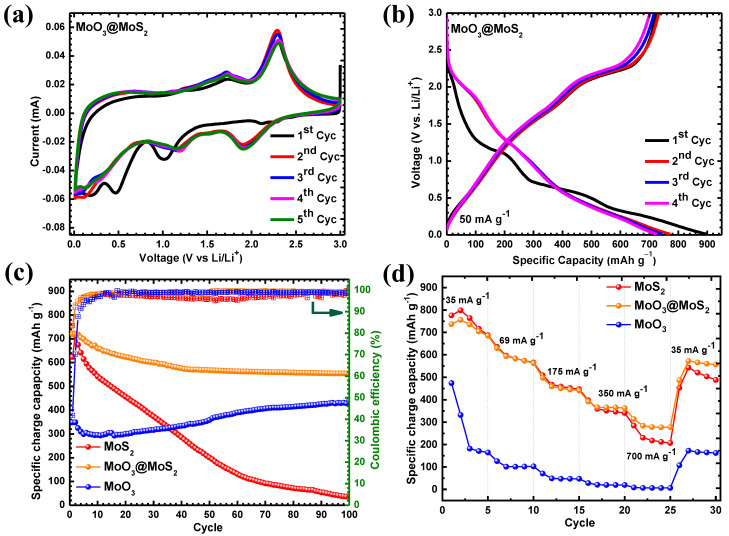
(**a**) CV and (**b**) discharge/charge profiles of MoO_3_@MoS_2_ between (0.01 and 3.00) V. (**c**) Comparative cycling performance at a current density of 50 mA g^−1^ and (**d**) rate performance of MoS_2_, MoO_3_@MoS_2_, and MoO_3_ anodes in LIBs.

The electrochemical performance of each sample was tested in the LIB half-cells. The cyclic voltammetry (CV) of the MoO_3_@MoS_2_ hybrid was measured in the voltage window of 0.01 to 3.0 V with a sweep speed of 0.1 mV s^−1^; the result is shown in Figure 6a. A reduction peak is located at 1.01 V, attributed to the Li-ion insertion between MoS_2_ layers and forming Li_x_MoS_2_. Another peak positioned at 0.46 V is linked to the conversion reaction of Li_x_MoS_2_ to (Mo + Li_2_S) [52]. These two reduction peaks shifted to a higher potential at about 1.91 and 1.15 V from the second cycle onwards. A shallow reduction peak at 0.1 V in the first cycle is the characteristic of solid electrolyte interface (SEI) formation. In the anodic scan, peaks located at 1.73 and 2.29 V are linked to the reverse conversion reaction of Mo + Li_2_S into MoS_2_, respectively [34]. The position of the oxidation peaks remains unchanged in the following cycles. Notably, a small irreversible peak at 2.11 V is found in the first cathodic scan, indicating Li^+^ intercalation into MoO_3_ crystals to generate Li_x_MoO_3_ [53]. Following the first cycle, the corresponding reduction peak did not occur, suggesting that the MoS_2_ is the predominant participant in the redox activity of the MoS_2_@MoO_3_ hybrid anode. Therefore, it seems that the main function of MoO_3_ is to stabilize MoS_2_. Further, the observed CV shows excellent reversibility from the 2nd to 5th cycles, which indicates superior cycling stability. The observed area under the curve of the MoO_3_@MoS_2_ composite is higher than that of its pristine components (shown in Figure S8a,b in SI), indicating enhanced capacity. Figure 6b shows the galvanostatic discharge/charge (GCD) curve of MoO_3_@MoS_2_ at the current density of 50 mA g^−1^; where the initial cycle reveals a remarkably high reversible (charge) capacity of ~721 mAh g^−1^. The observed capacity is close to the theoretical limit of MoO_3_@MoS_2_ (~737 mAh g^−1^). Based on the TGA data (Figure S5), we estimated the theoretical capacity as described in SI. Figure S9a–c shows the extended discharge/charge curves for MoS_2_, MoO_3_@MoS_2_, and MoO_3_. When comparing the GCD curves of the three samples, it can be seen that the MoO_3_@MoS_2_ hybrid outperforms the MoS_2_ and MoO_3_ counterparts in terms of cycling stability during 100 cycles, establishing its good stability as an active anode material for LIBs. Moreover, the similar trend of the charge/discharge curve for the hybrid anode from the second cycle indicates excellent electrochemical stability and conversion reversibility. In contrast, the charge/discharge capacities for the pristine MoS_2_ gradually deteriorated over the 100 cycles. Figure 6c further illustrates the difference in cyclability among MoS_2_, MoO_3_@MoS_2_, and MoO_3_ anodes. The MoO_3_@MoS_2_ anode possesses a higher initial charge (reversible) capacity than MoS_2_ and MoO_3_, with a Coulombic efficiency (CE) of 80.5, 83.5, and 41.5%, respectively. There is a significant decrease in capacity for the first few cycles of the MoO_3_@MoS_2_ anode. This phenomenon is commonly observed in the conversion-based anode materials, because during the discharge-charge process of the initial cycles, the SEI layer is not fully formed. This accounts for the low CE and capacity loss during initial cycles, as reported in previous studies [6,7,8,9,10,11,12,13,14,15,16,17,18,19,20]. The CV result described earlier also accords with this behavior. However, after a few cycles, the MoO_3_@MoS_2_ anode started to stabilize and the Coulombic efficiency improved, which is a marked difference from the pristine MoS_2_ or MoO_3_ anodes. The obtained result highlights the superior Li^+^ storage performance of the MoO_3_@MoS_2_ anode. In addition, the MoO_3_@MoS_2_ anode demonstrates a notably higher capacity (~564 mAh g^−1^) than that of MoS_2_ (~34 mAh g^−1^) and MoO_3_ (~434 mAh g^−1^) at the end of 100 cycles. Compared with an anode made of pure MoS_2_, the MoO_3_@MoS_2_ anode exhibits enhanced electrochemical properties because of the MoO_3_ passivation layer on the surface of the conductive MoS_2_. When we disassembled cells for MoS_2_ and MoO_3_@MoS_2_ electrodes after 30 cycles, we observed the active material residue on the lithium foil (counter electrode) for the MoS_2_ anode sample. Contrary to this, there was no active material found on lithium foil (counter electrode) in the hybrid anode sample (Figure S10). During the charge/discharge cycles, MoS_2_ and MoO_3_@MoS_2_ anodes undergo a steady conversion process, as evidenced by their Mo K-edge EXAFS spectra measured after the 50 cycles. The FT-EXAFS spectra peaks from their 50th cycle remain almost identical to those from their pristine state (Figure S11). Figure 6d and S12 show the rate capability of the MoO_3_@MoS_2_ hybrid and its pristine components (MoS_2_ and MoO_3_). As the current densities increase from 35 to 700 mA g^−1^, the capacities of the MoO_3_@MoS_2_ anode decrease; but when the current density is returned to its initial value (35 mA g^−1^), the active material retains a high-capacity value (~434 mAh g^−1^). The pure MoO_3_ shows very poor rate performance compared to MoS_2_, which is attributed to the poor conductive characteristics of the oxide material, as presented in Table 1.

**Figure 7:**
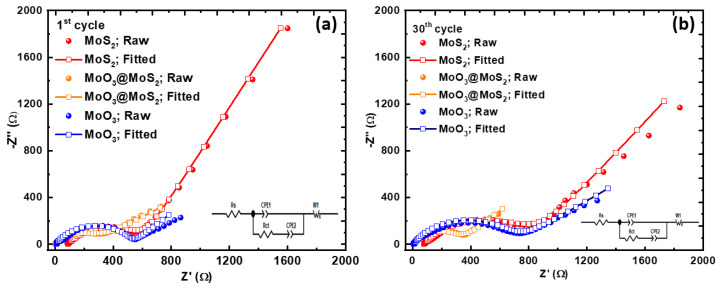
EIS profiles of MoS_2_, MoO_3_@MoS_2_, and MoO_3_ (**a**) after the 1st cycle, and (**b**) after the 30th cycle.

Figure 7a,b shows the electrochemical impedance spectroscopy (EIS) data with a fitted profile, measured after the 1st and 30th cycles, respectively. An equivalent electrical circuit is presented, where R_s_ is the solution resistance, R_ct_ is the charge transfer resistance, W is Warburg impedance, while the (CPE1 and CPE2) are the constant phase elements. Figure 7a clearly shows that the semi-circle of the MoO_3_@MoS_2_ hybrid anode Nyquist plot is much smaller than its counterparts (MoS_2_ and MoO_3_) after the 1st cycle. The calculated solution resistance (R_s_) and charge transfer resistance (R_ct_) of the hybrid anode are 2.392 and 284.308 Ω, respectively, which are lower than those of MoS_2_ and MoO_3_ (Table S3). Furthermore, after 30 cycles, the resistance of all the materials increases, but among them, MoO_3_@MoS_2_ shows much smaller resistivity. The hybrid anode material shows a lower R_ct_ value (304.539 Ω) among the three materials (Table S4) thanks to the chemically resistive nature of oxide, which protects the sulfide phase from the dissolution into the electrolyte.

## 4. Conclusions

A novel, in situ approach to the synthesis of a hybrid nanostructure has been successfully optimized. This involves a two-step process of a hydrothermal reaction with subsequent air annealing. The successful formation of the MoS_2_ core-MoO_3_ shell hybrid structure was verified by XRD, Raman, XPS, and XAS results. SEM and TEM studies reveal the morphology evolution with the growth of an oxide layer on the bulk sulfide, which facilitates stable cycling stability without sacrificing the high capacity and fast-charging performance. The MoO_3_@MoS_2_ hybrid anode delivered an excellent specific charge capacity of 721 mAh g^−1^, a good rate-capability of 278 mAh g^−1^ at 700 mA g^−1^, and the retained capacity was ~564 mAh g^−1^ after 100 cycles, with excellent (~99%) Coulombic efficiency. The outstanding performance of the hybrid anode results from the synergistic interaction between the passivating layer of MoO_3_, which eradicates the excessive dissolution of active material, and the conducting core of MoS_2_, which ensures smooth electronic conduction. The work presented in this paper provides a simple, two-step, facile method to synthesize different transition metal oxide/sulfide hybrid nanostructures.

## Figures and Tables

**Table 1:** Electrical conductivity measurement (by four-point probe) results.

Serial No.	Material	Average Electrical Conductivity (µS·cm^−^^2^)
01	MoS_2_	5.2442
02	MoO_3_@MoS_2_	0.6443
03	MoO_3_	0.0282

## Data Availability

The data presented in this study are available on request from the corresponding author.

## Supplementary Materials

The following supporting information can be downloaded at: https://www.mdpi.com/article/10.3390/nano12122008/s1, Figure S1: EDX spectrum of MoO_3_@MoS_2_; Figure S2: XRD of (a) MoS_2_ and (b) MoO_3_, including MoO_3_@MoS_2_, compared with their corresponding standard (PDF) data; Figure S3: Profile matching of HRPD data of (a) MoS_2_, (b) MoO_3_@MoS_2_, and (c) MoO_3_; Figure S4: XPS survey spectrum of MoS_2_, MoO_3_, and MoO_3_@MoS_2_; Figure S5: Thermogravimetric analysis (TGA) curve of as-synthesized MoS_2_ in air; Figure S6: HR-TEM image of the MoO_3_@MoS_2_ sample; Figure S7: (a–c) BET and (d) corresponding BJH profile of MoS_2_, MoO_3_, and MoO_3_@MoS_2_; Figure S8: CV curves of (a) MoS_2_ and (b) MoO_3_ anodes; Figure S9: GCD curves of the (a) MoS_2_, (b) MoO_3_@MoS_2_ and (c) MoO_3_; Figure S10: Photos of Li counter electrodes for the MoS_2_ and MoO_3_@MoS_2_ electrodes half-cells; Figure S11: Ex-situ Mo K-edge FT-EXAFS spectra of MoS_2_ and MoO_3_@MoS_2_ after 50 cycles; Figure S12: The galvanostatic voltage profiles (rate performance) of MoS_2_ and MoO_3_@MoS_2_ anodes at different current densities in LIBs; Table S1: Unit cell parameters of MoS_2_, MoO_3_@MoS_2_, and MoO_3_; Table S2: Result of BET analysis of MoS_2_, MoO_3_@MoS_2_, and MoO_3_; Table S3: Fitted solution resistance (R_s_) and charge transfer resistance (R_ct_) values of MoS_2_, MoO_3_@MoS_2_, and MoO_3_ after 1^st^ cycle; Table S4: Fitted solution resistance (R_s_) and charge transfer resistance (R_ct_) values of MoS_2_, MoO_3_@MoS_2_, and MoO_3_ after 30^th^ cycle; Table S5: Comparative table depicting electrochemical performance of selected TMO/TMS and/or carbon hybrids. References [33,36,54,55,56,57,58] were cited in Supplementary Materials.
