# Supplementary material for: MoO3@MoS2 Core-Shell Structured Hybrid Anode Materials for Lithium-Ion Batteries
Source: Nanomaterials (Basel). 2022 Jun 10;12(12):2008. doi: 10.3390/nano12122008 (PMC9229638; doi:10.3390/nano12122008)
Supplement: Supplementary file 1 [file nanomaterials-12-02008-s001.zip › nanomaterials-1745576-supplementary.pdf]

Supplementary Materials

$MoO_3@MoS_2$ Core-Shell Structured Hybrid Anode Materials for Lithium-Ion Batteries

Muhammad Faizan [1], Sajjad Hussain [2,3], Mobinul Islam [1,*], Ji-Young Kim [4], Daseul Han [1], Jee-Hwan Bae [4], Dhanasekaran Vikraman [5], Basit Ali [1], Saleem Abbas [6], Hyun-Seok Kim [5], Aditya Narayan Singh [1], Jongwan Jung [2,3] and Kyung-Wan Nam [1,*]

[1] Department of Energy & Materials Engineering, Dongguk University, Seoul 04620, Korea; faizijaff@gmail.com (M.F.); endend42@naver.com (D.H.); basitalikhan077@gmail.com (B.A.); aditya@dongguk.edu (A.N.S.)
[2] Hybrid Materials Center (HMC), Sejong University, Seoul 05006, Korea; shussainawan@gmail.com (S.H.); jwjung@sejong.ac.kr (J.J.)
[3] Department of Nanotechnology and Advanced Materials Engineering, Sejong University, Seoul 05006, Korea
[4] Advanced Analysis & Data Center, Korea Institute of Science and Technology (KIST), Seoul 02792, Korea; jykim1128@kist.re.kr (J.-Y.K.); jhbae@kist.re.kr (J.-H.B.)
[5] Division of Electronics and Electrical Engineering, Dongguk University-Seoul, Seoul 04620, Korea; v.j.dhanasekaran@gmail.com (D.V.); hyunseokk@dongguk.edu (H.-S.K.)
[6] Centre for Energy Storage Research, Korea Institute of Science and Technology (KIST), Seoul 02792, Korea; saleem.abbas203@gmail.com
* Correspondence: mobin85@dongguk.edu (M.I.); knam@dongguk.edu (K.-W.N.)

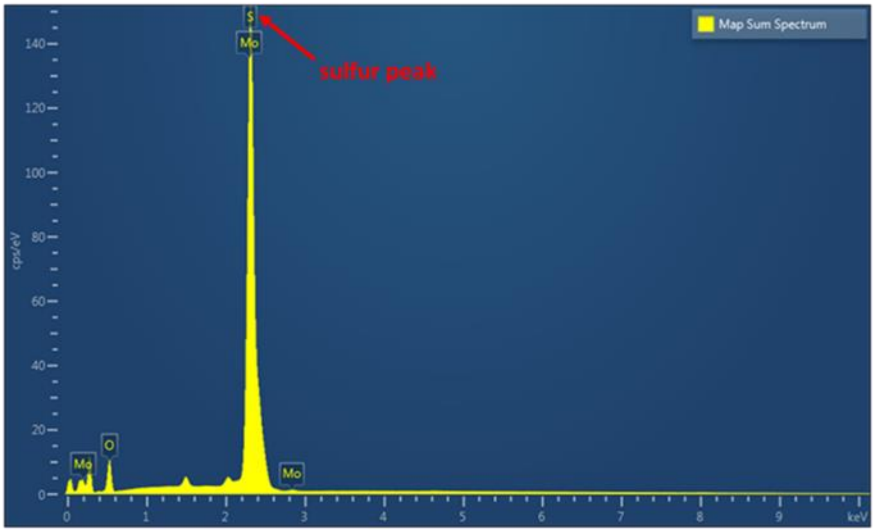

Figure S1. EDX spectrum of $MoO_3@MoS_2$.

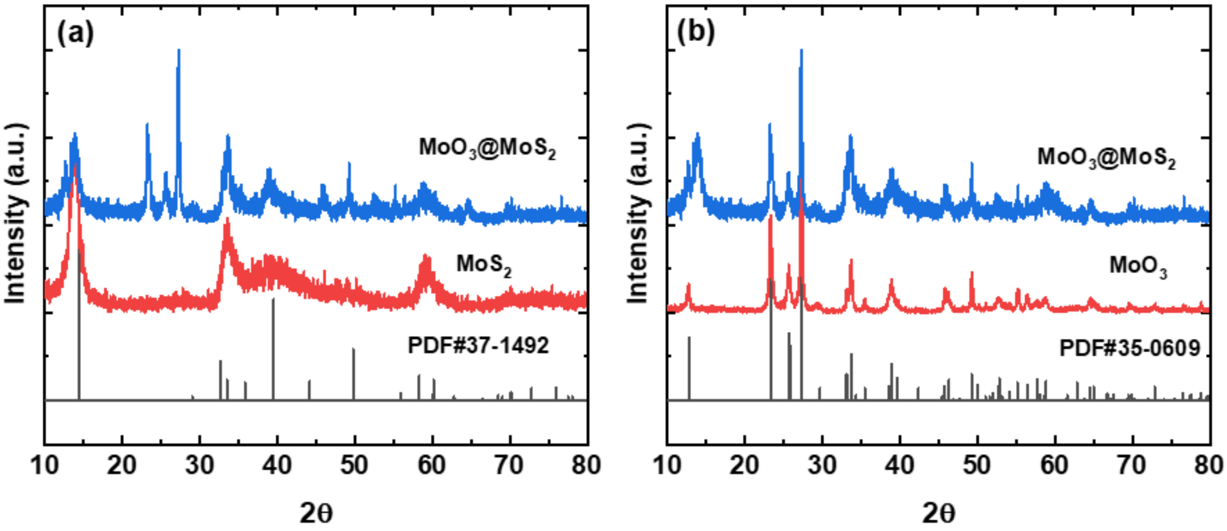

Figure S2. XRD of (a) MoS_2 and (b) MoO_3, including $MoO_3@MoS_2$, compared with their corresponding standard (PDF) data.

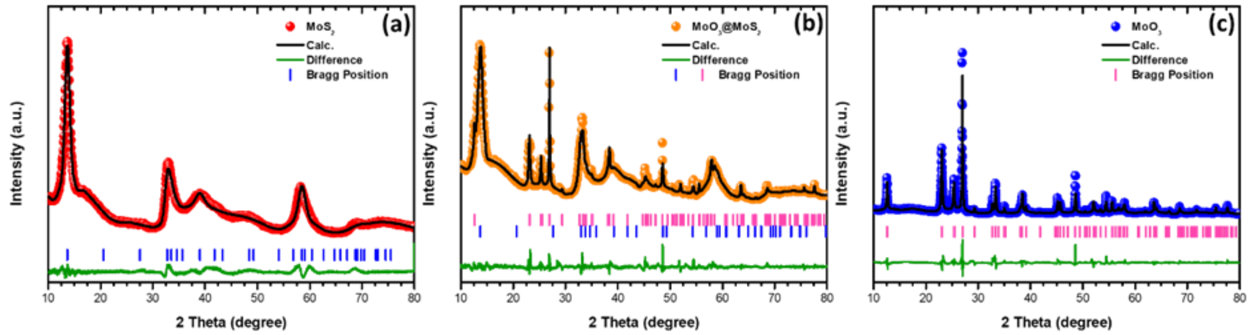

Figure S3. Profile matching of HRPD data of (a) MoS_2, (b) $MoO_3@MoS_2$, and (c) MoO_3.

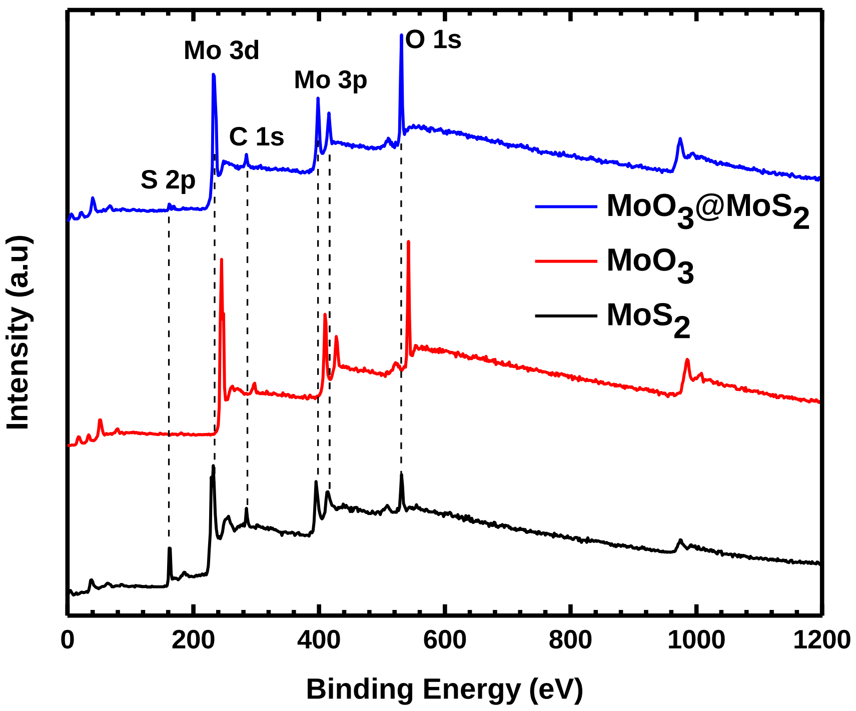

Figure S4. XPS survey spectrum of MoS_2, MoO_3, and $MoO_3@MoS_2$.

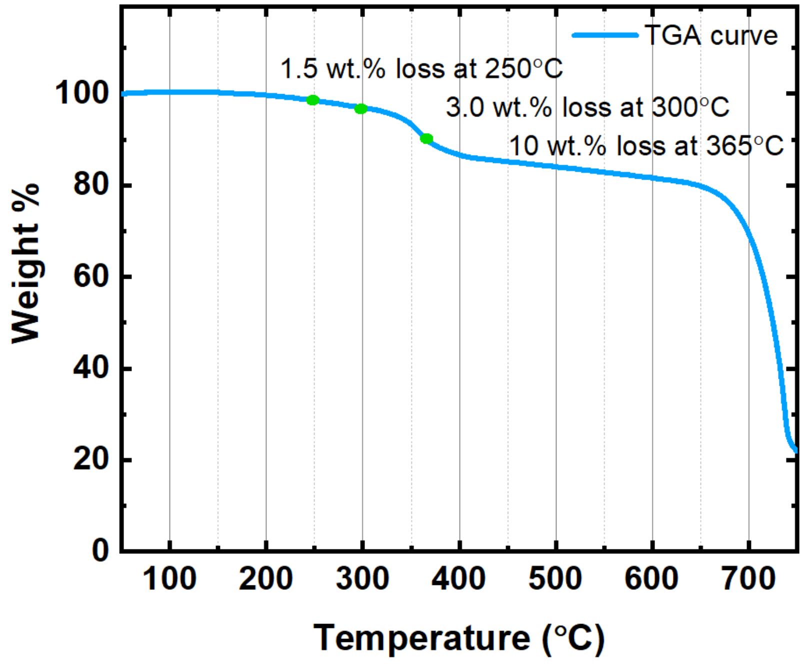

Figure S5. Thermogravimetric analysis (TGA) curve of as-synthesized MoS_2 in air.

<u>**Calculation of the ratio of MO_3 to MS_2 in the MoO_3@MoS_2.**</u>

MoS_2 (Mw: 160.07 g/mol) and MoO_3 (Mw: 143.94 g/mol)

If MoS_2 is fully decomposed to MoO_3,

MoO_3/MoS_2= 143.94 /160.07= 0.899 $\Rightarrow$ then 10 wt% decrease

From the TGA data, we observed 1.5 wt% loss after the oxidation process above 250 °C. Therefore, we can determine that MoS_2:MoO_3 ratio is approximately 8.5:1.5 in **MoO_3@MoS_2** hybrid based on the TGA data.

The **MoO_3@MoS_2** hybrid anode theoretical capacity (1 C= 737 mAh g^{-1}) calculated by the rule of mixtures of the two active materials:

($C_{MoO3@MoS2} = C_{MoS2} \times 0.85 + C_{MoO3} \times 0.15 = 670$ mAh $g^{-1} \times 0.85 + 1117$ mAh $g^{-1} \times 0.15$).

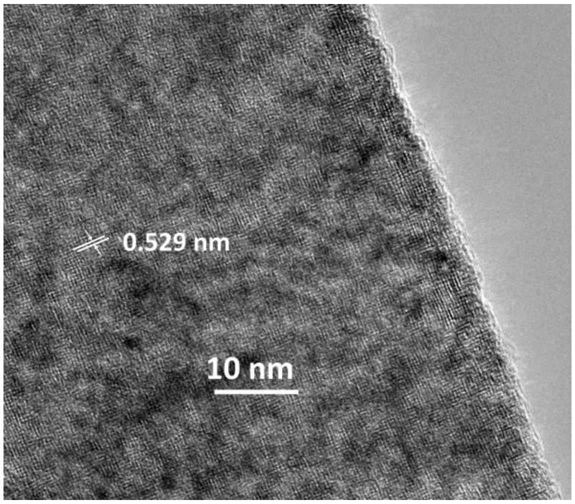

Figure S6: HR-TEM image of the $MoO_3@MoS_2$ sample.

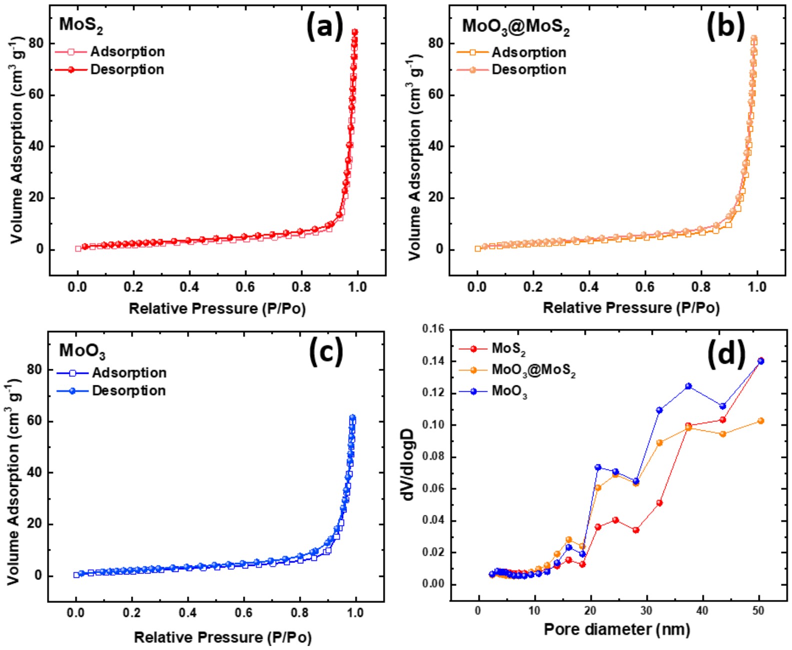

Figure S7. (a-c) BET and (d) corresponding BJH profile of MoS_2, MoO_3, and $MoO_3@MoS_2$.

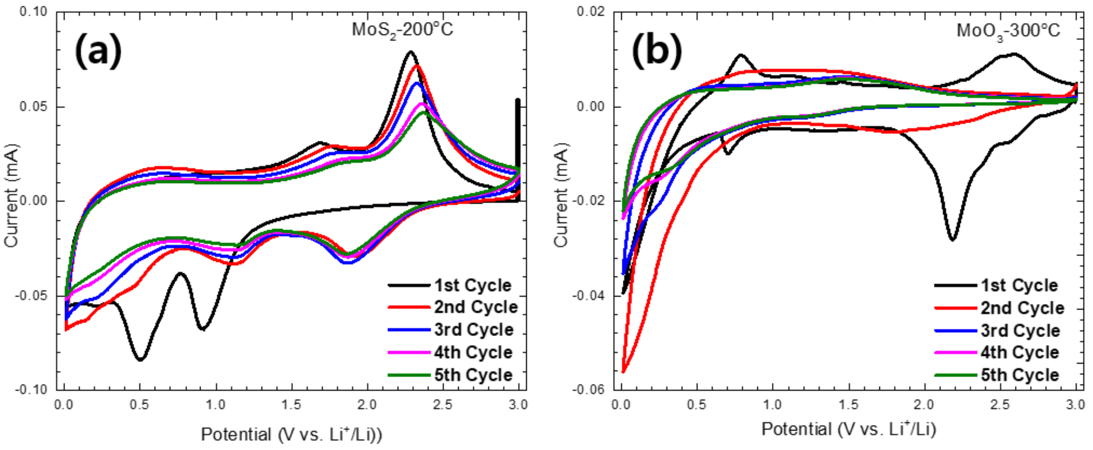

Figure S8. CV curves of (a) MoS_2 and (b) MoO_3 anodes.

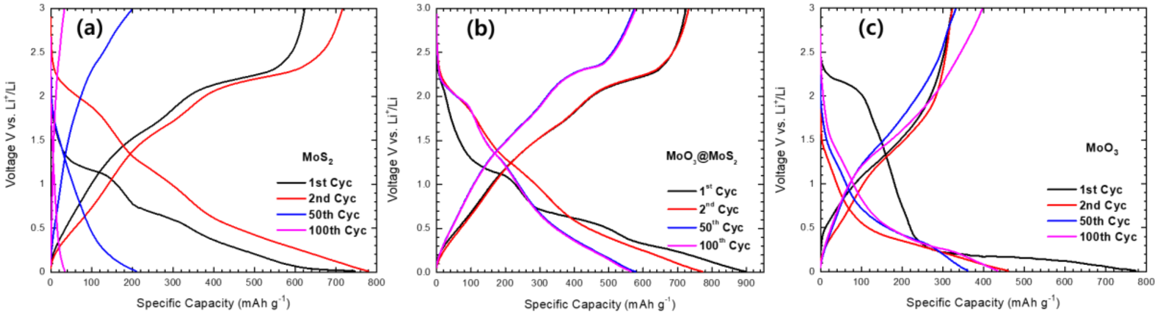

Figure S9. GCD curves of the (a) MoS_2, (b) $MoO_3@MoS_2$ and (c) MoO_3.

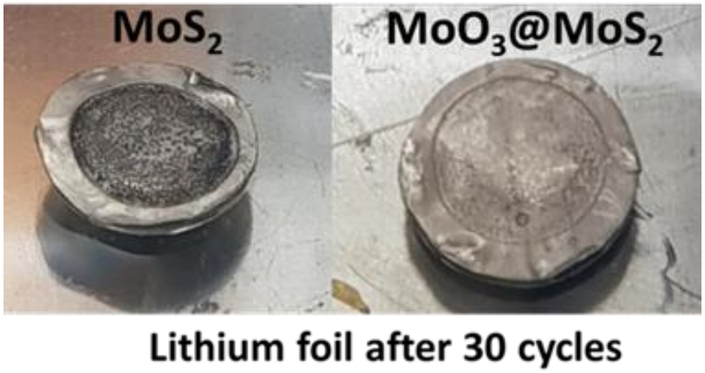

Figure S10. Photos of Li counter electrodes for the MoS_2 and $MoO_3@MoS_2$ electrodes half-cells.

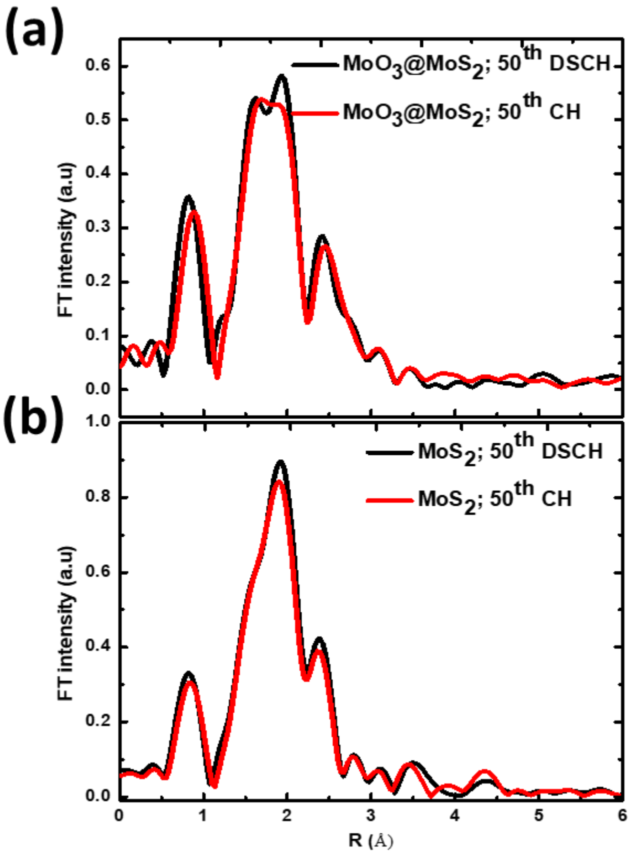

Figure S11. Ex-situ Mo k-edge FT-EXAFS spectra of MoS_2 and $MoO_3@MoS_2$ after 50 cycles.

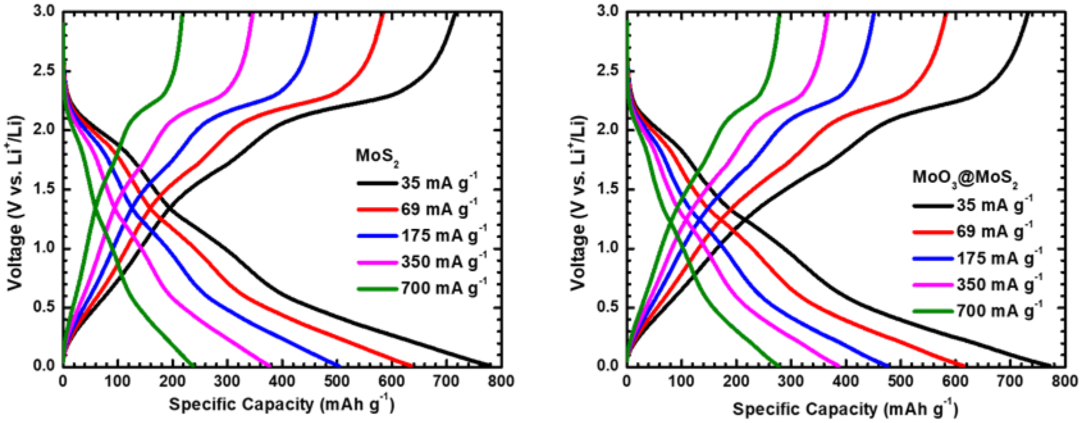

Figure S12. The galvanostatic voltage profiles (rate performance) of MoS_2 and $MoO_3@MoS_2$ anodes at different current densities in LIBs.

Table S1. Unit cell parameters of MoS_2, MoO_3@MoS_2, and MoO_3.

	MoS_2			MoO_3			
	**(a,b) Å**	**(c) Å**	**(v) $Å^3$**	**(a) Å**	**(b) Å**	**(c) Å**	**(v) $Å^3$**
**MoS_2**	3.1132	12.7912	107.3655				
**MoO_3@MoS_2**	3.0938	12.7677	105.8369	3.9518	13.8893	3.7001	203.0908
**MoO_3**				3.9615	13.8737	3.6981	203.254

Table S2. Result of BET analysis of MoS_2, MoO_3@MoS_2, and MoO_3.

**Sample**	**BET (m^2 g^{-1})**	**Pore volume (cm^3 g^{-1})**	**Mean pore diameter (nm)**
MoS_2	8.71	0.1011	46.423
MoO_3@MoS_2	8.42	0.129	61.61
MoO_3	9.85	0.1317	53.455

Table S3. Fitted solution resistance (R_s) and charge transfer resistance (R_{ct}) values of MoS_2, MoO_3@MoS_2, and MoO_3 after 1st cycle.

**Sample**	**R_s (Ω)**	**R_{ct} (Ω)**
MoS_2	82.26	473.24
MoO_3@MoS_2	2.392	284.308
MoO_3	6.656	518.44

Table S4. Fitted solution resistance (R_s) and charge transfer resistance (R_{ct}) values of MoS_2, MoO_3@MoS_2, and MoO_3 after 30th cycle.

**Sample**	**R_s (Ω)**	**R_{ct} (Ω)**
MoS_2	75.77	728.33
MoO_3@MoS_2	6.261	304.539
MoO_3	2.476	700

Table S5. Comparative table depicting electrochemical performance of selected TMO/TMS and/or carbon hybrids.

**Materials**	**Current density (mA g^{-1})**	**Cycle number**	**Reversible capacity (mAh g^{-1})**	**References**
SnS_2/SnO_2	100	80	520	[1]
MoO_3/SnS_2	100	100	504	[2]
TiO_2@MoS_2	100	100	544	[3]
VS_2@CNT	200	100	850	[4]
MoS_2/MoO_3/N-doped carbon	1000	350	887.5	[5]
$MoSe_2$@porous hollow carbon spheres (PHCS)	1000	100	681	[6]
Si@$Li_4Ti_5O_{12}$	500	150	883	[7]
**MoO_3@MoS_2**	**50**	**100**	**564**	**This work**

References

[1] K. Chang, W-X. Chen, H. Li, H. Li, Microwave-assisted synthesis of SnS_2/SnO_2 composites by L-cysteine and their electrochemical performances when used as anode materials of li-ion batteries, Electrochim. Acta, 56 (2011) 2856-2861.

[2] C. Hu, H. Shu, Z. Shen, T. Zhao, P. Liang, X. Chen, Hierarchical MoO_3/SnS_2 core-shell nanowires with enhanced electrochemical performance for lithium-ion batteries, Phys. Chem. Chem. Phys., 20 (2018) 17171-17179.

[3] X. Li, W. Li, M. Li, P. Cui, D. Chen, T. Gengenbach, L. Chu, H. Liu, G. Song, Glucose-assiste d synthesis of the hierarchical TiO_2 nanowire@MoS_2 nanosheet nanocomposite and its synergistic li thium storgae performance, J. Mater. Chem. A, 3 (2015) 2762-2769.

[4] X. Li, J. Fu, Y. Sun, M. Sun, S. Cheng, K. Chen, X. Yang, Q. Lou, T. Xu, Y. Shang, J. Xu, Q. Chen, C. Shan, Design and understanding of core/branch-structured VS_2 nanosheets@CNTs as high -performance anode materials for lithium-ion batteries, Nanoscale, 11 (2019) 13343-13353.

[5] K. Zhu, X. Wang, J. Liu, S. Li, H. Wang, L. Yang, S. Liu, T. Xie, Novel amorphous MoS_2/Mo O_3/nitrogen-doped carbon composite with excellent electrochemical performance for lithium ion bat teries and sodium ion batteries, ACS Sustain. Chem. Eng., 5 (2017) 8025-8034.

[6] X. Yang, Z. Zhang, Y. Fu, Q. Li, Porous hollow carbon spheres decorated with molybdenum diselenide nanosheets as anodes for highly reversible lithium and sodium storage, Nanoscale, 7 (2 015) 10198-10203.

[7] M. Liu, H. Gao, G. Hu, K. Zhu, H. Huang, Facile preparation of core-shell Si@$Li_4Ti_5O_{12}$ nanoc omposite as large-capacity lithium-ion battery anode, J. Energy Chem., 40 (2020) 89-98.
